# Feasibility Study of a Newly Developed Technology-Mediated Lifestyle Intervention for Overweight and Obese Young Adults

**DOI:** 10.3390/nu13082547

**Published:** 2021-07-26

**Authors:** Habiba I. Ali, Amita Attlee, Salma Alhebshi, Fadima Elmi, Ayesha S. Al Dhaheri, Lily Stojanovska, Najoua El Mesmoudi, Carine Platat

**Affiliations:** 1 Department of Nutrition and Health, College of Medicine and Health Sciences, United Arab Emirates University, Al Ain P.O. Box 15551, United Arab Emirates; amita.attlee@uaeu.ac.ae (A.A.); salma.alhebshi@uaeu.ac.ae (S.A.); Ayesha_aldhaheri@uaeu.ac.ae (A.S.A.D.); lily.stojanovska@uaeu.ac.ae (L.S.); najouam@uaeu.ac.ae (N.E.M.); PlatatCarine@uaeu.ac.ae (C.P.); 2 Independent Researcher, Al Ain P.O. Box 67258, United Arab Emirates; fa.elmi91@gmail.com

**Keywords:** technology-mediated, feasibility study, lifestyle intervention, mobile applications, obesity, young adults, United Arab Emirates

## Abstract

Background: Poor eating habits and sedentary lifestyle are common among young adults and increase the risk for chronic diseases later in life. Due to the widespread use of information technology among young adults, the Rashakaty (Fitness for Me) study aimed to develop and test the feasibility of a technology-based nutrition education intervention. This would support overweight and obese university students to achieve weight loss, enhance nutrition knowledge, and increase physical activity levels. Methods: We enrolled 246 participants in a 16-week non-randomized feasibility study with two arms: Rashakaty-Basic and Rashakaty-Enhanced. The intervention was guided by social cognitive theory and was delivered via a website and mobile apps. Results: Among the 161 participants who completed the endline assessments, there was no significant difference in weight loss between the two arms. However, waist circumference decreased more (*p* = 0.003) in the Rashakaty -Enhanced group. Additionally, changes in knowledge related to sources of nutrients (*p* < 0.001) and diet–disease relationships (*p* = 0.006) were significantly higher among the Rashakaty-Enhanced group. Rashakaty-Enhanced participants reported increased number of days spent on moderate physical activity (*p* = 0.013) and minutes walked (*p* < 0.001). Moreover, they also reported higher scores in social support from friends to reduce fat intake (*p* = 0.006) and from family and friends to increase physical activity (*p* = 0.001). Conclusions: The results of this feasibility study can assist in the development and implementation of future technology–mediated health promotion programs in the UAE, especially for young adults.

## 1. Introduction

Overweight, obesity, and associated chronic diseases are major public health concerns in the United Arab Emirates (UAE) [1,2,3]. Obesity prevalence in the UAE is above the global average; nearly one-third (30.0%) of adults, 39% females, and 27% males, are obese [4]. Obesity associated chronic diseases such as cardio-vascular disease, cancer and diabetes contribute to a 17% risk of premature deaths in UAE adults [4].

Weight gain in adults aged 18–30 years is associated with an almost 20 times greater risk of developing metabolic syndrome over the subsequent 15 years, compared to adults who maintain their weight over that period [5]. Studies indicate the prevalence of overweight and obesity among female university students in the UAE were 17.4% and 11.9%, respectively [6], and 28% and 14%, respectively [7]. Among adults from 187 countries, young adults aged 20–29 years had the lowest diet quality compared to any other age group [8]. Young adults, such as university students, are the most likely group not to meet the current nutritional recommendations. This is due to changes associated with moving away from home, busy schedules, and unhealthy eating patterns [9,10]. Sub-optimal dietary habits, including a high consumption of added sugars and low nutritional knowledge, have been reported in the UAE [11].

Basic nutrition knowledge is important to assist individuals in making healthy dietary choices and to improve eating behaviors [12]. However, previous studies conducted in the UAE focused only on specific aspects of nutritional knowledge among college students [7,11,13,14]. Therefore, there is a need to conduct a more comprehensive assessment of nutritional knowledge among university students in the UAE to assist in the development of targeted nutrition education programs.

Successful nutrition interventions should be based on evidence-based theoretical frameworks. Social cognitive theory (SCT) is well-recognized as a useful tool for identifying methods in which behavior can be changed [15,16,17,18]. Moreover, the SCT framework has been proposed as an appropriate nutrition intervention theoretical framework for university students [19]. There are three major constructs of SCT: self-efficacy, social support, and self-regulation strategies, such as setting reasonable health goals, and monitoring feedback regarding the effectiveness of strategies in meeting their goals [20].

Innovative weight management strategies are needed to tackle the obesity epidemic in the UAE. Interventions delivered primarily via information technology platforms, popular among young adults (18–35 years), should be considered. Recent meta-analysis and reviews have identified mobile technologies (such as SMS, apps) as being promising tools in facilitating weight loss [21,22].

Most of the studies that have evaluated the use of mobile technologies in weight management and changing nutrition lifestyle behaviors have been conducted in Western countries and the efficacy of such technology in nutrition research in the UAE has yet to be explored. Therefore, studies assessing the impact of new technologies on weight loss in countries undergoing nutrition transition, such as the UAE, are needed. To the best of our knowledge, there are no studies that explored the impact of nutrition intervention via new information technologies on weight loss, nutrition knowledge, and psychosocial factors influencing healthier food choices and regular physical activity in the UAE. The Fitness for Me (Rashakaty) study guided by the social cognitive theory aimed to:

(1) Design, test, and assess a new technology-based lifestyle intervention for overweight and obese students in two major universities in the UAE.
(2) Evaluate the impact of a lifestyle program delivered via information technology on weight loss and body composition (such as percent body fat and lean mass), nutrition knowledge, physical activity, perceived social support, and self-efficacy of overweight and obese university students.

## 2. Materials and Methods

### 2.1. Study Design

Fitness for Me (Rashakaty) is a non-randomized, two-arm feasibility study designed to develop and test nutrition education intervention, delivered via a website and mobile applications in overweight and obese university students in the United Arab Emirates (UAE). The study involved the development of a website, integrating educational material and mobile applications for monitoring diet and physical activity, and a 16-week feasibility trial to promote weight loss, improve food choices, and increase physical activity levels. The trial consisted of two arms: Rashakaty Basic (R-Basic) and Rashakaty Enhanced (R-Enhanced). R-Basic was implemented in the University of Sharjah and R-Enhanced in the United Arab Emirates University. Participants in the R-Basic arm received access to a static website that contained educational material on healthier eating and physical activity and questionnaires for completion at the baseline and at the end of the study period. The R-Enhanced intervention was based on social cognitive theory and employed a variety of behavior modification strategies, including self-monitoring, goal setting, self-efficacy, problem-solving, and social support to facilitate changes in diet and physical activity. The study was approved by the United Arab Emirates University Human Research Ethics Committee (Protocol # 33; 2014/2015). All procedures were in compliance with the principles of the Declaration of Helsinki. The trial was registered with ClinicalTrials.gov (accessed on 25 July 2021), NCT04919759.

### 2.2. Participants

Female students from two major universities (United Arab Emirates University and the University of Sharjah) in the UAE who were living either on or off-campus participated in the study. Overweight or obese students (BMI ≥ 25) aged between 18 and 35 years and who were current smartphone users were included. Those with past or planned weight loss surgery, pregnant or breastfeeding, had lost 7 kg or more in the past 3 months, were following any weight loss program for 3 months or more, taking any steroid or thyroid hormones treatment or any other medication that may affect body weight, were excluded. Students enrolled in the medical and nutrition programs were also excluded since one of the study outcomes was assessing changes in nutrition knowledge. 

### 2.3. Sample Size

To detect a mean difference of 1.97 kg with *p* = 0.05 and 80% power, assuming a standard deviation of 6.7 kg, 182 subjects were required (91 per group). When an attrition rate of 40% was considered, 255 subjects were needed (128 per group). This estimate was based on a 4-month trial among obese adults who reported an average weight loss difference between two intervention groups of 1.97 kg (95% confidence interval (CI) −3.60, −0.34) [23]. 

**Figure 1:**
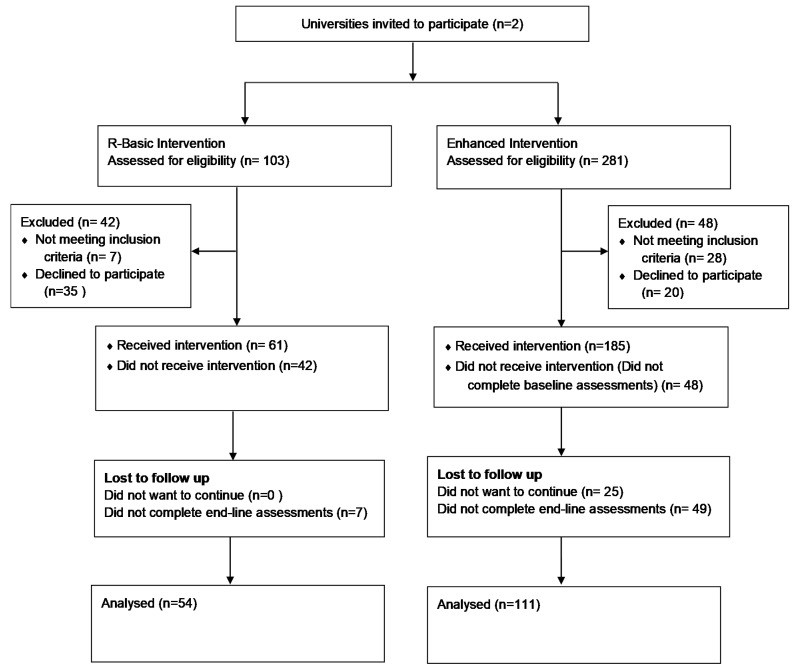
Flow of participants in the Fitness for Me program.

We aimed to recruit as many participants as possible within the first month of each of the two consecutive academic semesters (i.e., September 2016 and January 2017) to allow 16 weeks of intervention. The flow of participants in the program is presented in Figure 1.

### 2.4. Recruitment of Eligible Participants

A variety of means were used to reach overweight or obese female university students, including emails, university social media, and flyers in the educational and residential campuses. Potential participants were requested to register on the program website (www.Rashakaty.me; accessed on 30 October 2020). Research assistants scheduled appointments with the interested students using WhatsApp, phone calls, e-mails, and SMS text messages and invited them to the screening and recruitment sites on the campus of each university. During the introductory face-to-face meeting, the research assistants provided detailed information and answered questions about the study. Participants meeting the study inclusion criteria signed the consent form, and after the baseline anthropometric measurements were taken, the participants were requested to create an account on the program website.

### 2.5. Theoretical Framework of the Intervention

**Figure 2:**
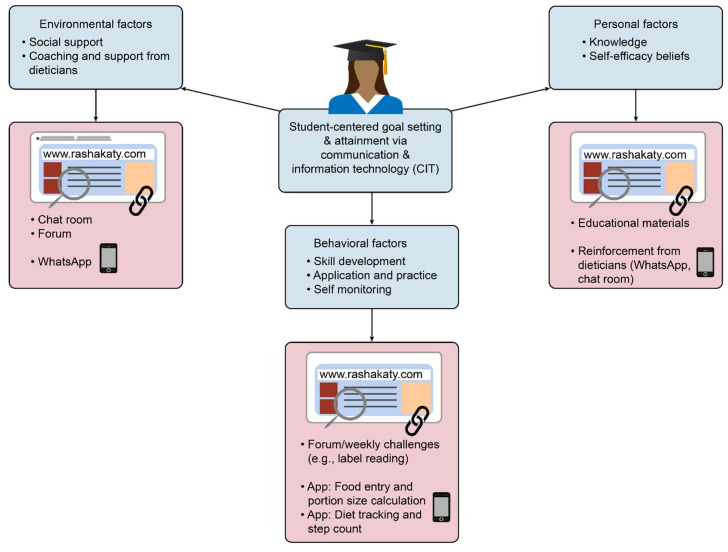
Application of social cognitive theory in R-Enhanced intervention.

The intervention to promote nutrition and other lifestyle behaviors was designed using social cognitive theory (SCT) [24,25,26]. SCT is helpful in understanding and predicting both the individual and group behaviors and identifying methods in which behavior can be modified. This provides a framework for designing, implementing, and evaluating programs [15]. The intervention in this study employed a variety of behavior modification and self-regulation strategies based on SCT, including self-monitoring, goal setting, self-efficacy, problem-solving, and social support to facilitate behavior changes (Figure 2 and Figure S1). 

### 2.6. Intervention Components

#### 2.6.1. Website

The Rashakaty website was developed as the main portal of the intervention. The components of the Rashakaty platform were designed to enable: (1) collection of data concerning diet and physical activity behaviors, (2) tracking of both diet and physical activity with mobile applications, (3) sharing educational materials, (4) providing on-line feedback and counseling, and (5) sharing news and challenges with participants. The website consisted of 6 tabs: the home tab, questionnaires tab, educational materials tab, get ready tab (to introduce self-monitoring apps), fitness+ tab (to enter step count results from PACER app), and the forum. The website forum was used to announce winners of the weekly challenges. Details of the study website tabs are given in Appendix A and its site map is presented as Supplementary Material (Figure S2). Table S1 shows a list of the weekly challenges announced in the forum.

#### 2.6.2. Self-Monitoring Strategies: Mobile Applications

Participants used mobile applications as self-monitoring tools in the R-Enhanced group. Participants who were interested in tracking food intake were given access to a commercial app (MyNetDiary). To adapt this app for cultural food preferences in the UAE, we incorporated 130 local composite dishes in the application’s database. Participants met with the nutritionist who assisted them in creating an account, selecting food portion sizes, and entering foods consumed in the app. Each participant was given a unique access code and a link to download the app on their phones. 

During the follow up, participants were encouraged to track their diet and enter all their food intake in the MyNetDiary (MND) app. Addressing questions or providing clarifications were conducted via WhatsApp, which was the preferred method of communication for the participants. Participants were also encouraged to monitor their physical activity by counting the number of steps and distance walked, using the PACER app. Nutritionists encouraged the participants to set goals and self-monitor their diet and physical activity. Nutritionists guided participants to meet their target goals, for example, a saturated fat intake of less than 10% of the daily calories. 

### 2.7. Measures

Data was collected upon enrollment in the study (baseline) and 16 weeks after enrollment in the study (endline). The following data was collected: anthropometric measurements (weight, height, Body Mass Index, waist circumference, and body composition), nutrition knowledge, physical activity levels, and psychosocial assessment (diet and physical activity related social support, and self-efficacy). All questionnaires were collected via online surveys hosted in the program’s website.

#### 2.7.1. Anthropometric Measurements

Data collection included height, weight, waist circumference, and body composition using bioelectric impedance (Tanita-BC420M, Tokyo, Japan). Height was measured using a portable stadiometer (Seca 213, Hamburg, Germany). All measurements were taken following standardized operating procedures prepared for the study. Participants were measured without shoes and in light indoor clothing. All the guidelines and instructions provided in the manufacturers’ manual were followed. A weight loss goal was discussed with each participant. It was recommended not to lose more than 0.5 kg/week and focus more on long-term adoption of healthier lifestyles.

#### 2.7.2. Nutrition Knowledge

We used the General Nutrition Awareness Questionnaire (GNKQ) validated by Parmenter and Wardle [27] on an adult population in the United Kingdom. The questionnaire was adapted to suit the study objectives and included 58 items divided into four main categories: (1) Awareness of dietary recommendations (11 items); (2) Knowledge of sources of nutrients (37 items); (3) Using the information to make dietary choices (5 items); and (4) Awareness of diet–disease relationships (5 items).

The questionnaire was translated into Arabic and reviewed for forward and backward translations. A panel of 10 bi-lingual (English and Arabic) academic staff reviewed the translated questionnaire for content clarity, difficulty, and accuracy. The adapted questionnaire was then pilot tested with 15 university students before using it in the study. Answers of each question item were scored by assigning one point for each correct response and zero points for an incorrect response. The scores of the items of each category were summed to obtain the participants’ category knowledge score.

#### 2.7.3. Physical Activity

The International Physical Activity Questionnaire (IPAQ)—Short Form was used to assess physical activity and inactivity of the participants [28]. This validated tool has been previously used in a national nutrition survey in the UAE [29], other surveys globally, and in the Arab Gulf Region [30,31].

#### 2.7.4. Psychosocial Questionnaire

We used the Health Beliefs Questionnaire, which was originally developed by Anderson and colleagues [16] after adaptation for university students in the UAE. Details of the adaptation process will be reported at a later stage. In summary, the questionnaire was found to be a reliable and useful tool to assess social support and self-efficacy for healthy eating and physical activity among Arabic-speaking female university students. The Cronbach’s alpha coefficient of the various sub-scales of the culturally adapted questionnaire ranged from 0.69 to 0.861, and the internal consistency of the various sub-scales ranged from 0.769 to 0.949 following 1 month of re-administration of the questionnaire.

### 2.8. Participant Feedback

Research nutritionists sought participant feedback about any challenges faced in using the program website or tracking their food intakes via the MND app. All participants who tracked their food intake were asked about challenges faced at least once in the study. This feedback was sought for further improvements of the intervention in the future. 

### 2.9. Statistical Analysis

Data was tested for normality with the Shapiro–Wilk test. Normally distributed variables are presented as mean ± SD, while skewed variables are presented as median (25–75%). Change in variables were calculated by subtracting baseline values from endline values. Statistical comparison between baseline and endline scores were performed using a paired t-test for normally distributed variables and the Wilcoxon signed-rank test for skewed variables. Statistical comparison of difference in the change between R-Basic and R-Enhanced were performed using the independent t-test for normally distributed variables and the Mann–Whitney U test for skewed variables. Comparison of Baseline values between R-Basic and R-Enhanced was performed using the Mann–Whitney U test. A chi-squared test was used to compare proportions of participants in the two groups who maintained or lost weight vs those who gained weight during the intervention.

The internal consistency of the items in the social support and self-efficacy subscales and the nutrition knowledge questionnaire categories were assessed using Cronbach’s alpha.

All analyses were performed using the Statistical Package for Social Sciences (IBM SPSS Statistics for Windows, v. 26); *p*-values < 0.05 were considered statistically significant.

## 3. Results

Two-hundred and forty-six female students from two major universities in the UAE were recruited in the Rashakaty study, out of which 165 (66.5%) completed the assessments at the end of the 16-week trial. There was no significant difference (*p* = −0.382) in baseline body weight between participants who completed the endline assessments and those who did not.

The mean age of the participants was 21.94 ± 2.03 years. Table 1 shows the anthropometric measures at recruitment (baseline) and at the end of the 16-week intervention (endline) as well as a comparison of changes of the two groups at baseline and at endline for each measure. 

### 3.1. Anthropometric Data

There were no significant changes in any of the parameters from baseline to endline in the Basic intervention, while there was a significant reduction in the median waist circumference from baseline to endline, from 91.00 (82.00–98.00) cm to 86.00 (81.17–89.67) cm, *p* < 0.001) in the Enhanced intervention. Similarly, there were significant decreases in BMI (*p* = 0.042), the percentage of body fat (*p* < 0.001), and the amount of body fat (*p* < 0.001) in the Enhanced intervention group (*p* < 0.001). Fat-free mass had increased (*p* = 0.043) after the 16-week intervention in the Enhanced intervention group (Table 2). At baseline, waist circumference of R-Enhanced was significantly higher than R-Basic (*p* = 0.009). A comparison of changes in anthropometric measures in the two groups showed a significant decrease in waist circumference of −2.00 (−6.00–0.00) cm among the Enhanced intervention group compared to the Basic intervention group (*p* = 0.003). On the other hand, there was no significant difference in weight loss between the Basic and Enhanced intervention participants. A comparison of the proportions of participants who maintained or lost weight vs gained weight between the two groups, using the chi-squared test, showed no significant difference (χ^2^ = 0.856).

### 3.2. Nutrition Knowledge

Table 2 shows scores in the four nutritional knowledge categories significantly increased among the R-Enhanced participants from their baseline levels (*p* < 0.001). On the other hand, among the R-Basic intervention participants there was only a significant increase in the knowledge scores of the *dietary recommendation* (*p* < 0.001) and the *choosing everyday foods* categories (*p* = 0.005). At baseline, participants in the R-Enhanced arm had significantly lower scores for sources of nutrients (*p* < 0.001), choosing everyday foods (*p* = 0.018) and diet–diseases relationships (*p* = 0.001), compared to R-Basic. The changes in nutrition knowledge categories between the two groups were significantly higher for *sources of nutrients* (*p* < 0.001), *choosing everyday foods* (*p* = 0.048), and the *diet–disease relationship* category (*p* = 0.006) in the R-Enhanced group. 

The internal consistency of the sub-categories was more than acceptable for *sources of nutrients* and *diet–disease relationships* (Cronbach’s alpha = 0.848 and 0.716, respectively) and extremely poor to poor for *dietary recommendations* and *choosing foods* (Cronbach’s alpha = 0.163 and 0.484, respectively). The overall reliability of the questionnaire was 0.823.

### 3.3. Physical Activity

Among the participants in the Basic intervention arm, there were no significant changes from baseline to endline in any of the physical activity levels tested except minutes of vigorous physical activity (*p* = 0.002) (Table 3). On the other hand, those in the Enhanced intervention arm had a significant increase from baseline minutes of vigorous physical activity (*p* = 0.025), days of moderate physical activity (*p* < 0.001 as well as minutes of moderate physical activity (*p* < 0.001) and in walking (*p* < 0.001) during the seven days preceding the interview. Moreover, there was a significant decrease in sitting time (*p* < 0.001) among the Enhanced intervention participants. 

Changes in physical activity showed significant difference in number of days (*p* = 0.005) and duration (*p* < 0.001) the participants in the Enhanced intervention arm engaged in vigorous physical activity compared to those in the Basic intervention arm. They also spent significantly more days (*p* = 0.013) performing moderate physical activity. Furthermore, participants in the Enhanced intervention had significant increase in minutes spent walking compared to those in the Basic intervention (*p* < 0.001).

### 3.4. Social Support and Self-Efficacy

As shown in Table 4, the mean scores of all the social support subscales for healthier food and physical activity had significantly increased from baseline to endline in the R-Enhanced arm (*p* < 0.001). Among the participants in the R-Basic group, there was a significant increase for support from friends in reducing fat intake (*p* = 0.032) and increasing fruit and vegetable intake (*p* = 0.001) from baseline to endline. 

At baseline, R-Basic participants reported higher scores for support from friends to reduce fat intake and increase fruit and vegetable intake (*p* = 0.025), as well as support to increase physical activity from family (*p* = 0.004) and friends (*p* = 0.029), compared to R-Enhanced. A comparison of changes in social support sub-scales related to healthier foods between the two intervention arms showed a significantly higher increase in scores for support from friends to reduce fat intake (*p* = 0.006) in the Enhanced intervention arm (Table 4). Moreover, the change in support from family and support from friends for physical activity were significantly higher among participants in the Enhanced intervention arm compared to those in the Basic intervention arm (*p* < 0.001 and *p* = 0.001, respectively).

There was a significant increase from baseline to endline in scores for self-efficacy to reduce sugar (*p* = 0.005) and self-efficacy to overcome barriers to physical activity (*p* = 0.012) in the Enhanced intervention arm (Table 5). There were no significant changes in any of the self-efficacy sub-scales among participants in the Basic Intervention arm. A comparison of changes between the two groups showed no significant differences in any of the self-efficacy subscales (Table 5).

### 3.5. Adapting the Program to the Need of the Participants

In order to design a website that is relevant for young female college students who are overweight or obese, we strived to make the website user-friendly. We used attractive images and designs, videos, quizzes, challenges, prizes, and a platform for discussion and sharing. The content of the website was tailored to evidence based on SCT (social cognitive theory) [15,26]. To maximize the effectiveness of the intervention, participants were also in continuous contact with the dietitians via WhatsApp, with the aim of receiving feedback and positive reinforcement from the nutritionist. The use of diet and physical activity mobile apps were used to facilitate behavior changes.

Participants cited a preference for the Arabic language in all materials on the website as well as shorter educational materials. However, it was not possible to redesign the website in Arabic due to time and budget constraints. We relied on the research assistants to explain the main principles of nutritional and physical activity and weight loss basic knowledge that were already available in the Nutrition Education tab of the program website. Research assistants also guided the participants in locating the relevant text or videos on the website as needed and addressed their questions. Participants stated that they would have preferred if all components of the Rashakaty intervention were delivered through mobile applications, instead of being partially delivered through a website.

The preferred means of communication for the participants was through WhatsApp. However, participants were encouraged to use a common forum provided on the website. Lack of time was the main limitation raised by participants due to their academic and other commitments. Moreover, participation in the program activities was low during the exam periods. This negatively affected the availability of the participants to complete the endline assessments due to their proximity to the final exam period. The participants faced challenges in estimating calories and the nutrient profiles of foods from restaurants. Moreover, due to many food choices for each food items in the MynetDiary app, they had difficulty in making decisions about the most appropriate choice that matched the food consumed. Some of the participants asked for an adapted meal plan for weight loss, a more traditional and restrictive way for losing weight. Preference for Arabic language in the program website and lack of time were the main reasons participants reported as the main reasons for requesting for a structured meal plan rather than using the MyNetDiary app. The program addressed the language issue by offering the online interactions with the participants (emails, WhatsApp, forum, and chatroom in the study website) in both Arabic and English languages, according to the preference of the participant. 

## 4. Discussion

Innovative weight management intervention methods are needed to tackle the obesity epidemic in the UAE. University students are typically a challenging group to engage in healthy behavior interventions due to their multiple commitments and time constraints. Information technology provides flexibility in terms of both data collection and engagement in the intervention programs. Both age and education were found to be important predictors for using mobile phones and apps [32]. A study conducted in Saudi Arabia found the highest use of weight-management apps was among women and especially highly educated overweight and obese women [33]. These findings support the potential application of mobile apps for weight management among female university students in the UAE. 

We designed an online nutrition education lifestyle intervention, “Rashakaty” (Fitness for Me)” to deliver weight loss intervention for overweight and obese female university students. Participants in both the Basic and Enhanced intervention were given online educational materials, with the aim of facilitating behavior changes through: (1) increasing awareness; (2) enhancing knowledge and skills; (3) creating a supportive environment for maintaining the adopted behavior changes. The delivery of the educational materials was guided by social cognitive theory. The intervention employed various behavior change self-regulation strategies, including goal setting and self-monitoring among participants in the Enhanced intervention arm.

Although participation in the Enhanced intervention arm led to greater improvements in physical activity, nutrition knowledge, and psychosocial-related scores, there was no significant improvement in weight loss compared to those in the Basic intervention. Collins and colleagues [34,35] did not find additional weight loss using an enhanced weight loss intervention involving combined personalized e-feedback and professional interaction over purely web-based 12- and 24-week basic intervention. Greater impact of the R-Enhanced compared to R-Basic intervention on some outcomes tested is consistent with previous research that has reported greater effectiveness of web-based interventions guided by health care providers compared to fully-automated interventions [36,37,38,39]. 

Self-regulatory behaviors, such as goal setting and self-monitoring play a crucial role in choosing healthier foods and engaging in regular physical activity for overweight and obese adults [40,41]. Moreover, interventions that involve social support, including family and friends, increase nutrition related self-efficacy and the adoption of healthier eating behaviors [16,40]. Participants in the Rashakaty Program were given access to mobile applications for tracking their food intake and physical activity levels and monitoring their progress in achieving their goals. They used a commercial diet tracking app. (MyNetDiary) and a step tracker app (PACER). Moreover, R-Enhanced had regular dietitian-participant interactions and feedback on dietary and physical activity as well as responses to their questions. Nutrition counseling through Rashakaty Program was easily adopted by university students. However, participants cited lack of time due to academic responsibilities and the need for website materials in the local language. Similar issues were identified in other Arab countries. The limited number of local foods [42] as well as the lack of apps in the Arabic language were reported as barriers to using weight management apps in Saudi Arabia [33,42]. 

Participation in the Rashakaty study led to a number of positive outcomes. Participants in the R-Enhanced group showed a significantly greater reduction in waist circumference compared to R-Basic (2 cm vs. 0.67 cm) after the 16-week intervention. However, the difference in change between the two groups may be partly explained by the significantly higher baseline waist circumference for the R-Enhanced group compared to the R-Basic group (91cm vs. 84.75 cm). Compared to R-Basic, participants in the R-Enhanced group reported a significantly greater increase in the number of days of vigorous activity and time spent walking, indicating the positive impact of using a mobile pedometer app. These finding are consistent with a previous meta-analysis which reported improved physical activity of young adults after participation in smartphone-based interventions [43]. Another positive outcome was the improvement of social support to increase the intake of fruits and vegetables observed in both groups, since a recent national survey indicated that 27% of those in the age group of 18–29 years consumed less than 5 servings of fruits and vegetables on an average day [44]. Our intervention could be used for potential future community programs. In contrast, support from family and friends to increase physical activity improved in the R-Enhanced group but not the R-Basic group, indicating that access to information through a static website may not be sufficient to improve social support. The lack of improvements in the self-efficacy measures in the R-Basic participants and improvements only in reducing sugar intake and overcoming barriers in the R-Enhanced group, indicate a greater effort may be required in changing these behaviors in future interventions.

The limitations of this study include the short duration of the intervention and the smaller sample of participants who completed the endline assessments due to a high attrition rate, and their unavailability due to their final exams at the time of the endline data collection. These factors might have contributed to the inability to detect clinically significant weight loss among participants in the intervention groups. Furthermore, participation in the Rashakaty program required a smart mobile phone; thus, those who did not use them were not included in the study. Another limitation is the existence of significant differences at baseline between the two groups for some of the outcomes tested. However, comparing baseline and endline values helped us evaluate the impact of R-Basic and R-Enhanced separately. Despite these limitations, to the best of our knowledge, this was the first study exploring the impact of nutritional intervention via new information technologies on weight loss and nutrition behaviors among young adults in the UAE. The lessons learned will be valuable in designing and implementing future, larger scale intervention studies with a longer duration and incorporating randomized controlled trials to test the long-term outcomes of technology-mediated lifestyle interventions for college students in the UAE. In addition, these studies should include a maintenance phase, add structured data collection on participant satisfaction with various intervention components, and develop a diet tracking tool for an Arabic-speaking population that contains locally relevant foods. Furthermore, considering the busy schedule of university students, interventions should focus on a single behavior change to reduce participant burden, such as increasing the intake of fruits and vegetables or self-monitoring physical activity by using mobile phone pedometers. In the absence of an updated nutrition knowledge assessment tool in Arabic, the original 1999 GNKQ version was adapted for this study. However, given the low scores in internal consistency in two of the four categories of the adapted questionnaire, the revised version of the General Nutrition Knowledge Questionnaire (GNKQ-R) which has been recently validated in the Arabic language in the UAE [45] will be useful for future studies in assessing nutritional knowledge of university students. 

## 5. Conclusions

Based on the lessons learned from the Rashakaty feasibility study, future interventions should consider the language use of both the website and educational materials in order to promote a greater program acceptance and involvement. Moreover, future interventions should be designed to be delivered solely via mobile applications since the use of a website was not particularly attractive or as accessible to participants as mobile applications were. Furthermore, weight management technologies that are culturally appropriate to the local context, including language and foods, are needed. This feasibility study can assist in the development and implementation of future technology-mediated health promotion programs in the UAE, especially for young adults.

## Figures and Tables

**Table 1:** Baseline and endline anthropometric measures in Rashakaty Basic (*n* = 54) and Rashakaty Enhanced *n* = 111) groups.

Variable	Group	Baseline *	Endline *	Change ^‡^
Waist circumference (cm)	R-Basic	84.75 ^a^ (80.75–91.25)	86.00 ^a^ (81.17–89.67)	−0.67 ^a^ (−3.08–3.67)
	R-Enhanced	91.00 ^a^ (82.00–98.00)	87.50 ^b^ (78.38–94.00)	−2.00 ^b^ (−6.00–0.00)
Weight (kg)	R-Basic	75.65 ^a^ (71.60–82.5)	76.93 ^a^ (72.30–82.68)	−0.37 ^a^ (−3.49–2.42)
	R-Enhanced	74.50 ^a^ (67.60–85.6)	72.80 ^b^ (67.00–85.6)	−0.20 ^a^ (−1.50–0.90)
BMI (kg/m^2^)	R-Basic	28.65 ^a^ (26.78–32.47)	29.36 ^a^ (27.76–31.64)	−0.14 ^a^ (−1.308–0.97)
	R-Enhanced	29.37 ^a^ (26.86–35.02)	29.43 ^b^ (26.57–35.11)	−0.08 ^a^ (−0.63–0.37)
Fat-free mass (kg)	R-Basic	47.95 ^a^ (46.30–50.15)	47.77 ^a^ (46.20–50.34)	−0.17 ^a^ (−1.27–0.78)
	R-Enhanced	47.10 ^a^ (44.50–49.20)	47.10 ^b^ (44.20–50.00)	0.20 ^a^ (−0.60–1.00)
Body fat percent (%)	R-Basic	36.10 ^a^ (33.90–40.13)	36.58 ^a^ (34.61–39.45)	−0.07 ^a^ (−1.80–1.59)
	R-Enhanced	36.50 ^a^ (33.30–42.30)	36.40 ^b^ (32.50–40.60)	−0.70 ^a^ (−1.70–0.60)
Body fat (kg)	R-Basic	27.00 ^a^ (23.03–32.48)	27.83 ^a^ (25.21–32.18)	−0.13 ^a^ (−2.77–1.77)
	R-Enhanced	27.00 ^a^ (22.80–36.50)	26.40 ^b^ (21.80–34.80)	−0.60 ^a^ (−1.90–0.50)

Values are presented as median (25–75%). Statistical comparisons between baseline and endline scores were performed using Wilcoxon signed-rank test. Different superscripts indicate significant difference (*p* < 0.05). * Statistical comparisons between baseline and endline scores were performed using Wilcoxon signed-rank test. ^‡^ Statistical comparisons of difference in change between R-Basic and R-Enhanced were performed using Mann–Whitney U test. Different superscripts indicate significant differences (*p* < 0.05).

**Table 2:** Baseline and endline nutrition knowledge in Rashakaty Basic (*n* = 54) and Rashakaty Enhanced (*n* = 111) groups.

Nutrition Knowledge Category ^†^	Group	Baseline *	Endline *	Change ^‡^
Dietary recommendations	R-Basic	7 **^a^** (4.75–8)	10 **^b^** (7–11)	3 **^a^** (1–4)
	R-Enhanced	7 **^a^** (6–8)	10 **^b^** (9–11)	3 **^a^** (2–4)
Sources of nutrients	R-Basic	24 **^a^** (17–27.25)	23 **^a^** (20–28)	1 **^a^** (−2.25–5.25)
	R-Enhanced	20 **^a^** (16–23)	26.5 **^b^** (24–29)	6 **^b^** (4.25–10)
Choosing everyday foods	R-Basic	3 **^a^** (2–3)	3 **^b^** (2–4)	0 **^a^** (0–1)
	R-Enhanced	2 **^a^** (1–3)	3 **^b^** (2–3.75)	0 **^b^** (0–1)
Diet–disease relationships	R-Basic	5 **^a^** (4–5)	5 **^a^** (4–5)	0 **^a^** (0–0)
	R-Enhanced	4 **^a^** (3–5)	5 **^b^** (4–5)	0 **^b^** (0–1)

^†^ Minimum and maximum score for each category: Dietary recommendations (0–11), Sources of nutrients (0–37), Choosing everyday foods (0–5), and Diet–disease relationships (0–5). Results are presented as median (25–75%) * Statistical comparisons between baseline and endline scores were performed using Wilcoxon signed-rank test. Different superscripts indicate significant difference (*p* < 0.05). ^‡^ Statistical comparisons of difference in change between R-Basic and R-Enhanced scores were performed using Mann–Whitney U test. Different superscripts indicate significant differences (*p* < 0.05).

**Table 3:** Physical activity levels in Rashakaty Basic (*n* = 54) and Rashakaty Enhanced (*n* = 111) groups.

Variable	Group	Baseline *	Endline *	Change ^‡^
Days of vigorous physical activity	R-Basic	0 ^a^ (0–2)	0 ^a^ (0–2)	0 ^a^ (−2–0)
	R-Enhanced	1 ^a^ (0–2)	1 ^a^ (1–2)	1 ^b^ (−1–1)
Minutes of vigorous physical activity	R-Basic	0 ^a^ (0–30)	0 ^b^ (0 -28.75)	0 ^a^ (0-5)
	R-Enhanced	10 ^a^ (0–42.5)	30 ^b^ (10–40)	5 ^b^ (0–26.25)
Days of moderate physical activity	R-Basic	0 ^a^ (0–2)	0 ^a^ (0–3)	0 ^a^ (−1–0.75)
	R-Enhanced	1 ^a^ (0–3)	3 ^b^ (0–4)	1 ^b^ (0–2)
Minutes of moderate physical activity	R-Basic	0 ^a^ (0–20)	0 ^a^ (0–20)	0 ^a^ (0–22.5)
	R-Enhanced	15 ^a^ (0–30)	15 ^b^ (0–45)	10 ^a^ (0–28.75)
Days of walking	R-Basic	4 ^a^ (2.5–6)	5 ^a^ (3.25–7)	1 ^a^ (0–2.75)
	R-Enhanced	7 ^a^ (4–7)	6 ^a^ (4–7)	0 ^a^ (−1–1)
Minutes of walking	R-Basic	30 ^a^ (20–40)	20 ^a^ (11.5–48.75)	−5 ^a^ (−20–8)
	R-Enhanced	25 ^a^ (15–36.25)	40 ^b^ (20–60)	15 ^b^ (0–35)
Sitting time (hours)	R-Basic	5 ^a^ (3–7)	8.5 ^a^ (4–14.25)	0 ^a^ (0–1)
	R-Enhanced	6 ^a^ (5–10)	5 ^b^ (3–7)	0 ^a^ (0–0.25)

Values are presented as median (25–75%) * Statistical comparisons between baseline and endline scores were performed using Wilcoxon signed-rank test. Different superscripts indicate significant difference (*p* < 0.05). ^‡^ Statistical comparisons of difference in change between R-Basic and R-Enhanced were performed using Mann–Whitney U test. Different superscripts indicate significant differences (*p* < 0.05).

**Table 4:** Baseline and endline perceived social support from family and friends for healthier foods and physical activity in Rashakaty Basic (*n* = 54) and Rashakaty Enhanced (*n* = 111) groups.

Variable ^†^	Group	Baseline *	Endline *	Change ^‡^
Perceived support from family to reduce fat intake	R-Basic	3.22 ^a^ (2.78–3.58)	3.44 ^a^ (3.00–3.78)	0.17 ^a^ (−0.19–0.56)
	R-Enhanced	3.11 ^a^ (2.67–3.44)	3.44 ^b^ (3.11–3.89)	0.33 ^a^ (0.00–0.78)
Perceived support from friends to reduce fat intake	R-Basic	3.11 ^a^ (2.64–3.33)	3.22 ^b^ (2.78–3.56)	0.22 ^a^ (−0.08–0.44)
	R-Enhanced	2.78 ^a^ (2.44–3.11)	3.22 ^b^ (2.89–3.75)	0.44 ^b^ (0–0.78)
Perceived support from family to increase fruit and vegetable intake	R-Basic	3.23 ^a^ (2.86–3.57)	3.57 ^a^ (2.89–3.71)	0.29 ^a^ (−0.29–0.57)
	R-Enhanced	3.00 ^a^ (2.71–3.43)	3.43 ^b^ (3–3.86)	0.29 ^a^ (0.00–0.71)
Perceived support from friends to increase fruit and vegetable intake	R-Basic	3.00 ^a^ (2.71–3.30)	3.14 ^b^ (2.75–3.57)	0.29 ^a^ (0.00–0.68)
	R-Enhanced	2.71 ^a^ (2.29–3.14)	3.29 ^b^ (2.86–3.68)	0.43 ^a^ (0.14–0.86)
Perceived support from family to increase physical activity	R-Basic	2.86 ^a^ (2.57–3.14)	2.86 ^a^ (2.57–3.14)	0.00 ^a^ (−0.29–0.29)
	R-Enhanced	2.57 ^a^ (2.14–3.00)	3.14 ^b^ (2.86–3.86)	0.57 ^b^ (0.00–1.29)
Perceived support from friends to increase physical activity	R-Basic	2.86 ^a^ (2.57–3.04)	3.00 ^a^ (2.57–3.25)	0.00 ^a^ (−0.25–0.43)
	R-Enhanced	2.71 ^a^ (2.29–3.00)	3.14 ^b^ (2.86–3.71)	0.43 ^b^ (0.00–1.14)

^†^ Perceived Social Support (Family and Friends) for healthier foods and physical activity sub-scales (1—Strongly disagree, 5—Strongly agree). Results are presented as median (25–75%) * Statistical comparison between baseline and endline scores using Wilcoxon signed-rank test. Different superscripts indicate significant difference (*p* < 0.05). ^‡^ Statistical comparison of difference in change between R-Basic and R-Enhanced scores using Mann–Whitney U test. Different superscripts indicate significant differences (*p* < 0.05).

**Table 5:** Perceived Self-Efficacy for choosing healthier foods and increasing physical activity in Rashakaty Basic (*n* = 54) and Rashakaty Enhanced *n* = 111) groups.

Variable ^†^	Group	Baseline *	Endline *	Change ^‡^
Perceived self-efficacy to reduce fat	R-Basic	75.00 ^a^ (66.07–86.61)	71.43 ^a^ (50.89–85.71)	−3.57 ^a^ (−16.96–9.82)
	R-Enhanced	71.43 ^a^ (57.14–82.14)	67.86 ^a^ (55.36–78.57)	0.00 ^a^ (−10.71–14.29)
Perceived self-efficacy to reduce sugar	R-Basic	58.33 ^a^ (41.67–75.00)	66.67 ^a^ (50–83.33)	0.00 ^a^ (−16.67–16.67)
	R-Enhanced	58.33 ^a^ (50.00–75.00)	66.67 ^b^ (50.00–75.00)	8.30 ^a^ (−8.33–20.83)
Perceived self-efficacy to increase fruits, vegetables and fiber	R-Basic	60.92 ± 21.46 ^a^	63.29 ± 22.43 ^a^	2.37 ± 22.77 ^a^
	R-Enhanced	59.10 ± 20.20 ^a^	63.41 ± 19.09 ^a^	4.31 ± 23.44 ^a^
Perceived self-efficacy to overcome barriers to physical activity	R-Basic	40.91 ^a^ (27.27–50)	45.45 ^a^ (27.27–59.09)	0.00 ^a^ (−13.64–17.05)
	R-Enhanced	40.91 ^a^ (31.82–59.09)	50.00 ^b^ (36.36–63.64)	4.55 ^a^ (−9.09–20.45)
Perceived self-efficacy to integrate physical activity into daily routine	R-Basic	57.69 ^a^ (50–69.23)	57.69 ^a^ (47.12–82.69)	0.00 ^a^ (−11.54–15.38)
	R-Enhanced	69.23 ^a^ (53.85–80.77)	69.23 ^a^ (50.96–84.62)	0.00 ^a^ (−11.54–11.54)

^†^ Self-Efficacy for healthier Foods and physical activity subscales (0—Certain I cannot, 50—Somewhat certain I can, 100—Certain I can). Results are presented as mean ± SD for normally distributed variables and median (25–75%) for skewed variables. * Statistical comparison between baseline and endline scores using paired t-test for normally distributed variables and Wilcoxon signed-rank test for skewed variables. Different superscripts indicate significant difference (*p* < 0.05). ^‡^ Statistical comparison of difference in change between R-Basic and R-Enhanced scores using the independent t-test for normally distributed variables and Mann–Whitney U test for skewed variables. Different superscripts indicate significant differences (*p* < 0.05).

## Data Availability

The dataset supporting the findings of the reported results is available from the corresponding author on reasonable request (HIA).

## Supplementary Materials

The following are available online at https://www.mdpi.com/article/10.3390/nu13082547/s1: Figure S1. Rashakaty.me Site map; Figure S2. Behavioral change strategies applied in Rashakaty Enhanced intervention; Table S1. List of challenges posted in the website forum.

## Appendix A. Rashakaty Website Tabs

The various tabs of the Rashakaty Program website are presented in Figure S1. The access to the tabs was made sequential to ensure that the participants followed a unified path to the website information and completed the necessary steps of each component of the website. The website components available to the participants in both groups (R-Basic and R-Enhanced) were the Questionnaire tab and Education Material.

Home tab: The Home tab contained information about the: (1) program, (2) research team, (3) inclusion/exclusion criteria, (4) consent form, (5) rewards for joining the program, (6) registration form, and (7) chat box with nutritionist.

Questionnaires tab: The study questionnaires were available from the Questionnaire Tab. Participants in both universities were asked to complete the following three questionnaires: (1) Psychosocial questionnaire (The Health Beliefs Questionnaire Adapted for Arabic-speaking young adults), which consisted of two scales: Perceived Healthier Foods Social Support and Step count Social Support and Perceived Healthier Foods Efficacy and Step Count Efficacy; (2) Nutrition Awareness among Adults Questionnaire; (3) International Physical Activity Questionnaire (IPAQ-Short Form). These questionnaires were administered using Google forms in bilingual (English and Arabic) versions to suit the preference of the participants.

Once the participants created their accounts in the Rashakaty website, the first tab directed them to complete all the pre-intervention questionnaires. Once completed, the system automatically allowed them to move to the next tab, the Nutrition Education tab.

Educational materials tab: This tab contained a series of evidence-based articles, videos, and pdf documents to guide the participants on the principles of healthy weight loss and in three-step wise behavior-building strategies:

Step 1: Increasing awareness of the importance of a healthy lifestyle.

Step 2: Practical ways to achieve weight loss and proven tips and techniques that can help people stay fit and achieve their target weight.

Step 3: Staying on track and keeping the acquired skills to facilitate maintenance of the acquired healthy behaviors.

Upon reviewing the educational materials, participants were advised to complete a short quiz. The following website tabs were accessible only to participants in the R-Enhanced group.

Get ready tab: The apps for diet monitoring (MyNetDiary app) and physical activity tracking (PACER App) were introduced in this Tab. Participants in the R-Enhanced arm were invited to download these apps and use them.

Fitness+ tab: Using this tab, participants were requested to enter the results of their step count using the PACER App.

The fifth tab was the Forum, where the participants were encouraged to share their success stories, questions, join discussions, find support, and participate in the weekly challenges with peers and research assistants. Motivational quotes were added to this website tab, mimicking the Facebook page in allowing adding images, commenting on them, and liking them. The forum was also used to post the weekly challenges. The weekly challenges posted for a period of 12 weeks were a fun way to create encouragement and improve adherence to the program. Participants were informed the weekly that the weekly challenge would be posted every Friday to encourage participation. The first five winners each received a USD 50 gift voucher as a reward.
