# Supplementary material for: Feasibility Study of a Newly Developed Technology-Mediated Lifestyle Intervention for Overweight and Obese Young Adults
Source: Nutrients. 2021 Jul 26;13(8):2547. doi: 10.3390/nu13082547 (PMC8399959; doi:10.3390/nu13082547)
Supplement: Supplementary file 1 [file nutrients-13-02547-s001.zip › nutrients-1259991-supplementary/Supplementary material/Table S1. List of challenges posted in the website forum.pdf]

Table S1. List of challenges posted in the website forum

Time	Day of activation on website	Day of deactivation on website	Description	Conditions to win
Week 1	Friday	Monday	Today's challenge:<br>For this challenge, we want you to go to the nearest market and take pictures of two food products that contain less than 150 calories	The 5 first participants to submit the right answer
Week 2	Sunday	Wednesday	Today's challenge:<br>For this challenge, we want you to show a picture of yourself with at least one friend or relative being physically active	The 5 first participants to submit the right answer
Week 3	Friday	Monday	Today's challenge: For this challenge, we want you to go to the nearest mart and take pictures of 1 juice with the label "without added sugar", one food item with the label "no trans-fat" and one food item with the label " Low Salt".	The 5 first participants to submit the right answer
Week 4	Saturday	Tuesday	Today's challenge:<br><br>For this challenge, we want you to take a	The 5 first participants to submit the right answer

			screenshot of your app "Moves" showing the exact distance (in meters) between the food court and the library	
Week 5	Sunday	Wed	Today's challenge:<br><br>By now, you should be familiar with healthy eating. Build a healthy plate when you eat at the food court and show it to us. Tip: We have shown you how to do this in Nutrition 101.	The 5 first participants to submit the right answer
Week 6	Friday	Monday	Today's challenge:<br><br>For this challenge, we want you to take us outside the University and show us a nice place where we can be active.	The 5 first participants to submit the right answer
Week 7	Saturday	Tuesday	Today's challenge:<br><br>For today's challenge, we are interested in local foods. Based on the knowledge you have acquired in this program, what do you think is the healthiest traditional Emirati food? Show us the pictures, OR show the responses in pictures	The 5 first participants to submit the right answer
Week 8	Sunday	Wed	Today's challenge:<br><br>For this challenge, show us how active you are; Take a screenshot of your app "Moves" showing the number of steps you did today. The highest number of steps will receive a free gift.	The 5 first participants to submit the right answer

Week 9	Friday	Monday	Today’s challenge:<br><br>For today's challenge, we want you to tell us what is in your opinion, the healthiest choice available in the vending machines on campus. Show us in pictures.	The 5 first participants to submit the right answer
Week 10	Sunday	Wed	Today’s challenge:<br><br>Do you feel like you have no time to exercise? Be creative and show an easy exercise you can do while in class and get our free gift.	The 5 first participants to submit the right answer
Week 11	Saturday	Tuesday	Today’s challenge:<br>We want to have lunch at the healthiest restaurant in Al Ain. In your opinion, which one we should go to? Show us in pictures.	The 5 first participants to submit the right answer
Week 12	Saturday	Tuesday	Today’s challenge:<br><br>For today's challenge, show us a picture of 3 common tools you can use in a room at home to do exercise without any equipment like a treadmill.	The 5 first participants to submit the right answer
