# Supplementary figures and images for: Feasibility Study of a Newly Developed Technology-Mediated Lifestyle Intervention for Overweight and Obese Young Adults
Source: Nutrients. 2021 Jul 26;13(8):2547. doi: 10.3390/nu13082547 (PMC8399959; doi:10.3390/nu13082547)

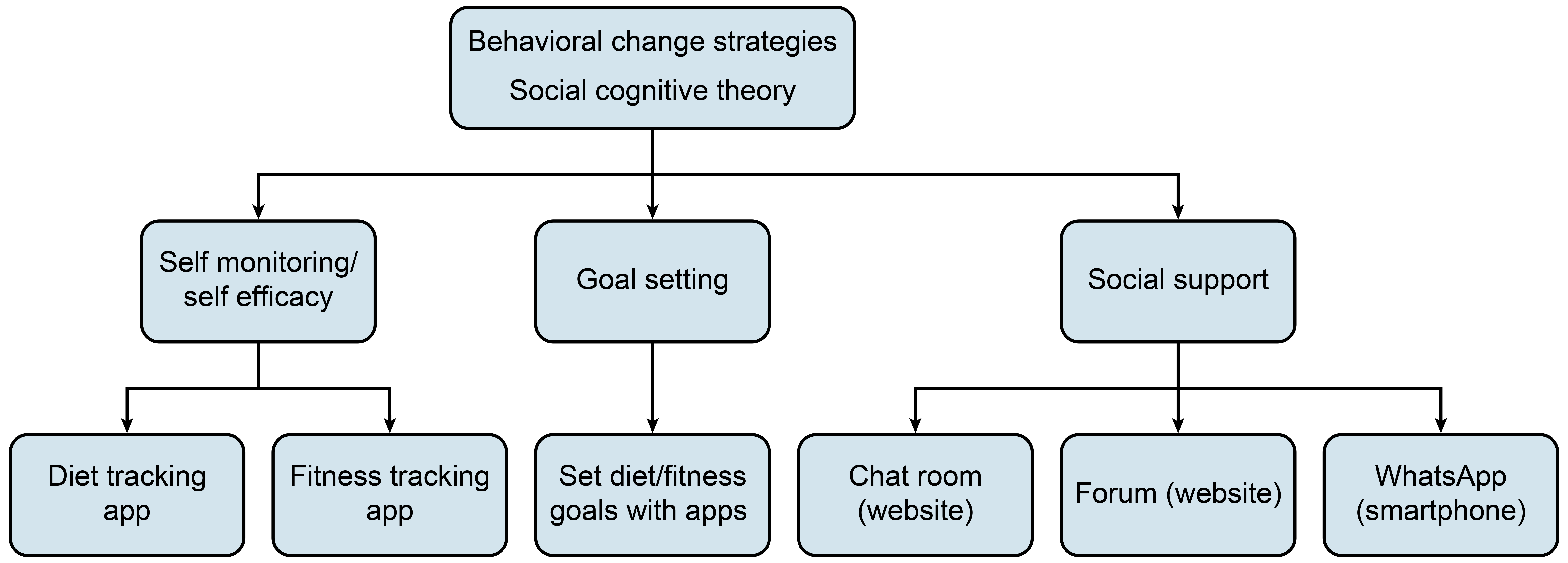

Supplement: Supplementary file 1 [file nutrients-13-02547-s001.zip › nutrients-1259991-supplementary/Supplementary material/Figure S1, Behavioral change strategies.tif]
